# Impact of Safety Culture on Safety Performance; Mediating Role of Psychosocial Hazard: An Integrated Modelling Approach

**DOI:** 10.3390/ijerph18168568

**Published:** 2021-08-13

**Authors:** Gehad Mohammed Ahmed Naji, Ahmad Shahrul Nizam Isha, Mysara Eissa Mohyaldinn, Stavroula Leka, Muhammad Shoaib Saleem, Syed Mohamed Nasir Bin Syed Abd Rahman, Mohammed Alzoraiki

**Affiliations:** 1 Department of Management & Humanities, Universiti Teknologi PETRONAS, Seri Iskandar 32610, Perak, Malaysia; shahrul.nizam@utp.edu.my (A.S.N.I.); sh.saleem87@gmail.com (M.S.S.); 2 Department of Petroleum Engineering, Universiti Teknologi PETRONAS, Seri Iskandar 32610, Perak, Malaysia; mysara.eissa@utp.edu.my; 3 Cork University Business School, University College Cork, T12 K8AF Cork, Ireland; stavroula.leka@ucc.ie; 4 Petronas Group Technology Solutions, Bandar Baru Bangi 43000, Selangor, Malaysia; syedmohdnasir@petronas.com; 5 Department of Technology Management and Business, Universiti Tun Hussein Onn Malaysia, Parit Raja 86400, Johor, Malaysia; alzoraiki88@gmail.com

**Keywords:** safety culture, psychosocial hazards, safety performance, oil and gas, Malaysia

## Abstract

We conceptualize that safety culture (SC) has a positive impact on employee’s safety performance by reducing their psychosocial hazards. A higher level of safety culture environment reduces psychosocial hazards by improving employee’s performance toward safety concerns. The purpose of this study was to evaluate how psychosocial hazard mediates the relationship between safety culture and safety performance. Data were collected from 380 production employees in three states of Malaysia from the upstream oil and gas sector. Structural equation modeling was implemented to test the suggested hypotheses. The proposed model was evaluated using structural equation modeling. A stratified sampling with a Likert 5-point scale was used to distribute the questionnaires. Furthermore, the proposed model was tested using the simulation of the structural equation and partial. According to our findings, all hypotheses were significant. A review of prior studies was used to select the items of the dimension for the data collection. Safety culture was assessed with psychosocial hazard to determine its direct and indirect impact on safety performance. Results suggest that to enhance safety performance (leading and lagging), psychosocial concerns in the workplace environments should be taken into consideration by employees. In addition, the findings showed that the psychosocial hazard fully mediates the relationship between safety culture and safety performance.

## 1. Introduction

Over the last 100 years, the emphasis on occupational safety at work has considerably helped to save thousands of lives. Fatalities and accidents at work were quite prevalent in the early 1900s [1]. For example, one early occupational accident study revealed that more than 500 workers per year died with a further 1500 severe and non-fatal incidents having occurred in Allegheny County Pennsylvania. According to Hoffmann, et al. [2], Malaysia’s oil and gas industry is a major contributor to the national economy. Malaysia’s national oil company is no exception. However, even with these improvements, occupational safety at work remains a major problem. According to the Social Security Organization of Malaysia (SOCSO), it announced that the latest number of reported industrial accidents was 35,304 in 2016, which increased by 1357 cases or 3.84% to a total of 36,661 in 2017 [3,4].

Yang et al. [5] specifically discussed safety performance as a comprehensive safety management system with output controlled and measured by safety management, safety organizations, safety training activities, safety equipment, incident investigations, safety training evaluations, and accident statistics measures. A safety performance assessment enables organizations to assess leadership efficiency, but many safety performance definitions challenge safety performance evaluations. Safety efficiency includes testing policy understanding, onshore visits, and tours, attending monthly security meetings, occupational health plans and programs, and closing off a large proportion of correctives [6]. Traditionally, oil and gas companies use lagging indices to thoroughly evaluate the safety performance of employees [7], such as Total Recordable Incident Rate (TRIR) and the severity rate (SR) of work security and health administration (OSHA). In the workplace, the TRIR is a calculation of the reported injuries per 100 employees per year. As a result, the TRIR multiplies the highest figures of injuries throughout the year by 200,000; the average working hours are simply split by that amount per year. The security Rate (SR), in contrast, is a ratio that defines the number of days lost compared to the number of events [8,9].

Management is not short of safety procedures and processes; however, compliance remains the issue that needs to be addressed immediately. Three central characteristics common to all organizations are people’s behaviors, organizational structure, and organizational processes. Research has shown that focusing on a well-established structure, organizational processes, and strict rules and regulations alone does not guarantee a successful health safety and environment (HSE) performance [10]. Avoiding this tunnel-vision is vital in a workplace that involves an enormous number of people, a high-hazard work environment, and the potential for loss of life [11]. Effective employees enable the organization to function properly and influence others who work in the same organization to fulfill the required goals. However, the ‘softer’ human resource concerns have too often been accorded little attention [12], and there is a need to understand better the factors that influence employees’ safety performances.

## 2. Literature Review

### 2.1. Safety Culture

According to Yorio et al. [13] the idea of safety culture was raised after the 1986 Chernobyl incident. As stated by [14,15], there is no universally accepted definition for safety culture as several interpretations have been assigned to safety culture by many researchers since its inception. Regardless of this, certain elements make it possible to constitute a definition for safety culture as a set of shared values, and members’ safety perception, attitude and their behavior concerning safety in an organization as well as organizational policies, procedures, and practices pertinent to the enhancement of measures against possible risks and hazards being the priority [15]. Cox and Cox [16] also highlighted the concept of safety culture by including collective beliefs, perceptions, and attitudes of the workforce in the pursuit of safety. Therefore, different studies have defined safety culture as shown in Table 1. The dimensions of safety culture will be applied to assist employees’ stress and safety performance, such as management commitment, work environment, and involvement. Management commitment increases employees’ skills, which helps to save lives, reduce stress, and prevent injuries in any organization [17]. Involvement or work demands on workers associated with limited management and work-life balance activities have been found to increase the rate of stress [18]. Consequently, this research work clarifies the understanding and development of safety attitudes that would reduce accidents at the workplace [19], as stress causes depression, anxiety, and anger, which can result in huge costs that would be a burden to the organization [20,21]. According to the literature, psychosocial risks and hazards have been aggravated and have become more intense, and it is organizational legislation and structure that protects workers from such hazards or at least helps minimize their effect [22]. Therefore, the culture of the organization, i.e., safety culture, is expected to play a vital role in the mitigation of psychosocial hazards. We thus formed Hypothesis 1 to test the association between safety culture and psychosocial hazards.

**Hypothesis** **1.** *There is a significant relationship between safety culture and psychosocial hazard*.

### 2.2. The Dimension of Safety Culture

#### 2.2.1. Management Commitment

Commitment to management means being engaged in a mannered behavior that supports other staff to accomplish the determined goals [29]. In general, measurements can be assessed in two different ways: direct questions are asked by managers [30] or their commitment behaviors are monitored. According to Joung et al. [30], some managers acknowledge that they are not committed to safety rules when asked, while such behavior requires obvious proof for safety commitment.

#### 2.2.2. Work Environment

The work environment requires procedures and policies be put in place to ensure the health and safety of staff in the workplace. The key policy is the identification of hazards and controls based on standards set by the government, and performing safety training and education for employees [31]. Moreover, a healthy and safe work environment has professional and legal responsibilities to afford staff with a workplace that is free of hazards which may cause serious physical injury or death, and to preserve safe and healthy working conditions for their staff.

#### 2.2.3. Involvement

Work involvement refers to supporting and promoting employees to participate and be consulted on health and safety matters in their workplaces. Setting such a goal is necessary, as involving employees can have an encouraging effect on health and safety performance [32]. In addition, work involvement combines management, and health and safety representatives in inspections, investigations, and risk assessments.

### 2.3. Psychosocial Hazards

Chirico et al. [33] defined psychosocial hazards as all occupational hazards that have a direct or indirect effect on the employees’ psychological being and their capability to contribute in a work environment amongst other individuals [34]. Psychosocial hazards are connected to the organization, project, and management of the work, as well as the social context and economics of the work, and are tied to psychological, psychiatric, and physical injuries or illnesses. Similarly, the International Labour Organization [35] alluded to psychosocial hazards as being two sides of a coin: the relations amongst job content, the organization, and management of the work, as well as environmental and organizational conditions, are on one side, and on the other side, are the individuals’ proficiencies and desires [36]. Thus, these interactions engender a strong influence over the health of employees through their perceptions and experiences [37]. In effect, these psychosocial hazards are now unique challenges to organizations/managers, and thus these hazards ought to be effectively managed. In recent times, workplace psychosocial hazards have been recognized as a major contemporary challenge to occupational health and safety, and they include but are not limited to workplace problems such as work stress, workplace violence, and workplace bullying [38,39].

Psychosocial hazards and risks were developed according to the International Labor Organization (ILO) definition from 1986. The interaction among work environment, work organization, job content, workers’ capacities, needs, cultures, and personnel consideration are all considered psychosocial factors that have a great influence on work performance and job satisfaction. These variable interactions are acknowledged as having a potentially hazardous effect on workers’ health [40]. There are nine categories identified by stress researchers who grouped psychosocial risks to job content, workload and work pace, working hours, participation and control, career development, status, payments, role in the organization, interpersonal relations, organizational culture, and home-work interface [41,42]. Work-related stress is the harmful physical and emotional response caused by an imbalance between the perceived demands and the perceived resources and abilities of a person to cope with those demands. Based on previous studies, [43,44] reported that there is a relationship between psychosocial hazards and safety performance. Thus, we formed hypothesis 02 to test the relationship between psychosocial hazards and safety performance.

**Hypothesis** **2.** *There is a significant relationship between psychosocial hazards and safety performance.*

### 2.4. Safety Performance

One of the most important elements to enhance the efficiency of oil and gas projects is increasing the performance of the safety activities. While significant consideration is on the use and creation of inventive indicators for safety performance, contractor’s safety has historically been assessed and controlled by using lagging indicators [6]. Organizational safety performance assessment helps organizations assess management effectiveness, but the safety performance assessment is challenged by various classifications.

Safety performance as the global output of the security management of an enterprise can be conceptualized by six factors: safety equipment, safety measures, safety management, accident investigation statistics, safety training, and safety organization [5]. Bunner et al. [45] presented safety performance models comprising performance elements, performance determinants, and performance backgrounds. The performance history was determined at the individual and organizational levels. The individual-level includes performance roles, skill, experience, and personality. The organizational level includes an organization’s atmosphere, which assigns meaning to individuals and values the characteristics of the work environment. There are three success elements: awareness, capacity, and motivation. The performance components define the actual behaviors of the people at work, such as compliance with health and safety performance [46].

#### 2.4.1. Lagging and Leading Metrics

The lagging indicator measures the influence of safety on workplace accidents after they happen. Specific staff behaviors and daily activities are analyzed using leading indicators [47]. This approach allows managers and employees to alter habits before accidents or incidents happen. Thus, leading indicators accurately work as a warning tool for both managers and employees to act before the occurrence of any property damage, personnel injuries, or even any harm. The available lagging indicators could not provide satisfactory information about real reasons for workplace incidents [6]. This failure requires companies to perform further research and studies to figure out the actual reasons for health and safety incidents. The leading indicators allow employees and managers to have an effective system and process to track employee’s safety performance. Such a tracking system rapidly identifies workplace safety failure and examines the root causes by emphasizing staff activities and attitudes [48].

**Figure 1:**
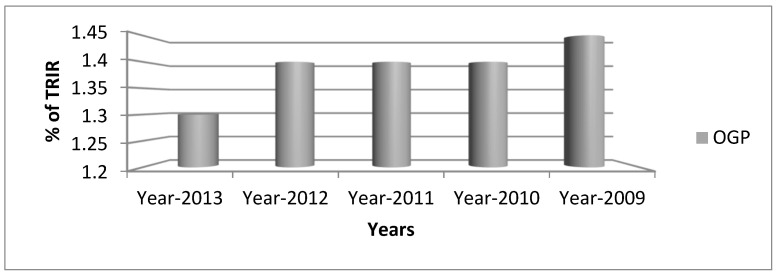
TRIR Analysis indicators in the safety field.

In case these tests indicate that any component of the safety program is defective or even deteriorating, adaptive improvement and even swift mitigation measures need to be implemented to enhance the safety program and influence safety outcomes [49]. According to the US Bureau of labor statistics, safety performance efficiency is historically assessed by lagging measures in oil and gas companies [50], including Total Recordable Incident Rate (TRIR) as shown in Figure 1 variation of lagging indicators in the safety field. TRIR in the period between 2009 and 2013 had irregular patterns that were generally above the limit of the Oil and Gas Producers (OGP). Therefore, the section demonstrates insufficient safety performance [51]. Regarding the anomalies of leading indicators, the frequency has an inconsistent pattern and the same can be observed in Figure 1.

Lagging indicators are considered as variables related to incidents of failure or past events [52]. Because lagging indicators give rise to the reaction after the accident, safety performance lagging indicators are dependent on previously obtained results [47]. Leading or upstream indicators are measures that enable you to predict and anticipate. They provide a precursor to any safety cycle deterioration, allowing early intervention by managers. Lagging or downstream interventions are factors for accidents and situations that have occurred (or have not occurred) [53]. As opposed to that, Leading Safety Performance Indicators (SPIs) have been employed and tracked since at least 1985, when the international association of oil and gas producers (OGP) started databasing its global member companies on safety incident statistics, providing analysis, measurement, and identification of areas and activities that occupational health and safety should focus on to achieve the greatest performance improvements and reduce the number of incidents that occur [54].

Fast forward and today’s success in safety is now measured using the same tools and techniques that are essential to quality control measures of other organizational measures. Control charts, running charts, and Pareto charts can be used to track and monitor established trends and safety performance, and to measure system performance against accepted tolerances [55]. Empirical metrics cannot be derived from the leading indicators, however, reasonable predictions can be made from them for future performance levels [47]. Hence, leading indicators correspond to lagging indicators [6,56]. As suggested by previous studies, a positive safety culture is required to improve safety performance [57,58]. Thus, based on the above discussion, the following hypothesis is developed.

**Hypothesis** **3.** *There is a significant relationship between safety culture and safety performance*.

#### 2.4.2. Psychosocial Hazard Mediates the Relationship between Safety Culture and Its Impact on Employees Performance

The previous studies have recognized that psychosocial hazards could be influenced by the efficiency of the safety culture amongst the workers [59]. As aforementioned, researchers have found that an effective safety culture is essential to engage employers in safety matters, which then affects the level of safety performance [60]. According to Kortum [61], research has acknowledged the positive impacts of the safety culture establishing workplace psychosocial hazards. Although one of the best ways to improve the safety of psychosocial risk is to optimize safety culture, employees usually tend to be reticent about encountering risky situations [62] which might decrease the level of safety psychosocial hazards in the organization.

Thus, when the workers in the organization are having safety information being disseminated well, this will be reflected in the level of safety psychosocial hazards. According to Lunt [63], safety psychosocial risk is related to employee’s perceptions of the safety conditions at the workplace. A more positive safety psychosocial risk has been found to maintain the participation of the workers in safety-related issues, for which the influence of a worker’s perception of the psychosocial safety risk will determine the safety-related behavior of the worker [64]. As noted in previous studies, a positive level of safety psychosocial risk will encourage and enhance the worker’s safety performance at work [65]. Hence, the researcher of the current study believes that an effective safety culture will enhance the level of safety psychosocial risk, which will lead to an increase of the safety performance of the workers. Although evidence has attested to a significant direct relationship between safety culture and safety performance, this research believes that such a relationship could be refined with the inclusion of psychosocial hazard in a mediator role between safety culture and safety performance. According to the outcomes of previous studies [66,67], when construction employees in the upstream oil and gas sector in Malaysia perceive a positive safety climate or safety culture at work, they will focus their efforts on completing the work rather than on safety practices, as they face the prospect of being laid off. As a result, they may break more safety standards, resulting in a higher accident and injury rate in the workplace environment. Therefore, it is hypothesized that:

**Hypothesis** **4.** *A psychosocial hazard mediates the relationship between safety culture (management commitment, work environment, and involvement) and safety performance (leading and lagging indicators)*.

**Figure 2:**
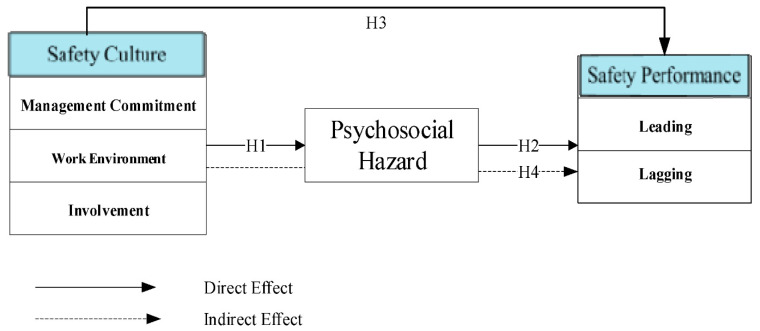
Conceptual framework.

The current study follows the same argument based on the social exchange theory by [68], to create the conceptual framework, stating that employees who work in an environment with positive safety culture are more likely to perceive their contributions and feedback as being valued by management. Hence, by being beneficial to their well-being and showing support for safety, the employees tend to alter or improve their behavior by proactively participating in safety-related activities. This theory suggested that, as studied, the social behavior in the interaction of two parties implement a cost-benefit analysis to determine risks and benefits. Therefore, the social exchange theory says that if the costs of the relationship are higher than the rewards, this could lead to issues. Thus, this theory reflects the relationship between the safety culture and safety performance through the psychosocial hazard. As a result, we proposed the following conceptual framework based on this theory (see Figure 2).

## 3. Methods and Materials

### 3.1. Participants and Procedures

The survey included productions and operational employees of nine different oil and gas companies operating in three Malaysian states (Sabah, Sarawak, Trengganu). Because the nature of their employment exposes them to significant safety problems and dangers, production and operational employees (mostly involved in oil extraction and processing) were included [44]. Pipe-liners, electricians, and chemical foremen, as well as material specialists, are among the production and operational employees [69]. An analysis of variance was used to see if there was a difference between the two groups before testing the hypothesis. In terms of the study’s primary variables, the results indicated that there is no difference between these groups. Operation and production professionals in the oil and gas business have a unique job description that required them to be particularly cautious in their work because a single mistake might result in a psychosocial hazard. According to the literature, such employees are particularly vulnerable to accidents [70,71]. Because of their continual exposure to toxic and hazardous materials, oil and gas personnel are classified as a “safety-sensitive” industry in the safety literature [72].

The questionnaires have been distributed among the employees in the upstream oil and gas sector of Malaysia. During the completion of the questionnaire, the principal researcher remained in the organization to answer any questions that respondents might have. A total of 380 responses were received from the employees in the upstream oil and gas sector. Thus, the total sample size was N = 380. The majority of the respondents were Malay 95 (95%), while 5 (5%) were Chinese. The largest number of respondents, 210 (55.263%), were between 30 and 39 years old; 90 (23.68%) of the respondents were between 40 and 49 years old; 50 (13.16%) of the respondents were between 20 and 29 years old; and 30 (7.89%) of the respondents, the smallest number, were between 50 and 59 years old. Table 2 shows the demographic characteristics of the respondents.

### 3.2. Measures

#### 3.2.1. Safety Culture Scale (SCS)

The safety culture scale (SCS) was adopted by [73,74] to measure the level of safety culture in this research. It is composed of three dimensions, namely management commitment (6 items), work environment (5 items), and involvement (5 items). The authors [73,74] measured the reliability of the safety culture scale for each dimension, and found the values of the reliability to be as follows: management commitment (0.896), work environment (0.872), and involvement (0.922). Therefore, the measurement of the items used a five-point Likert scale (1 = Never, 2 = Seldom, 3 = Neutral, 4 = Often, 5 = Always).

#### 3.2.2. Management Commitment (MC)

Management commitment (MC) was measured by [73], using 6-items, which include the following: (1) “ in my workplace, the management acts quickly to correct safety problems”, (2) “management acts decisively when a safety concern is raised”, (3) “in my workplace, management turns a blind eye to safety issues”, (4) “corrective action is always taken when I tell management about unsafe practices”, (5) “in my workplace, managers/supervisors show interest in my safety”, and (6) “management acts only after accidents have occurred”.

#### 3.2.3. Work Environment (WE)

Work environment (WE) was measured by [73], using 5-items, which include the following: (1) “ operational targets often conflict with safety measures”, (2) “ sometimes I am not given enough time to get the job done safely”, (3) “sometimes conditions here hinder my ability to work safely”, (4) “there are always enough people beside me to get the job done safely”, and (5) “I cannot always get the equipment I need to do the job safely”.

#### 3.2.4. Involvement (INV)

Involvement (INV) was measured by [74], using 5-items, which include the following: (1) “management always welcomes opinion from employees before making final decisions on safety-related matters”, (2) “my company has safety committees consisting of representatives of management and employees”, (3) “management promotes employees involvement in safety-related matters”, (4) “management consults with employees regularly about workplace health and safety issues”, and (5) “ employees do not sincerely participate in identifying safety problems”.

#### 3.2.5. Psychosocial Hazards (PSH)

Psychosocial hazards (PSH) were measured using the Copenhagen Psychosocial Questionnaire (CPQ) [75], which consists of five items and was used to assess psychosocial hazards at the workplace, where the value of the reliability has been found (0.864). This scale’s purpose is to assist the employees to reduce the psychosocial hazards at the workplace. Items include: (1) “is your workload unevenly distributed so it piles up?”, (2) “how often do you not have time to complete all your work tasks?”, (3) “do you get behind with your work?”, (4) “do you have enough time for your work tasks?”, and (5) “do you have to work very fast?”.

#### 3.2.6. Safety Performance Scale (SPS)

Safety performance scale (SPS) was developed by [76,77] to measure the level of safety performance. The reliability value was found to be (0.907). It is composed of two dimension measurement indicators, namely, leading indicator (4 items) and lagging indicators (5 items). SPS was developed by [76,77], who measured the reliability for the safety performance scale for each dimension. The values of the reliability we found are as follows: leading (0.864) and lagging (0.849).

#### 3.2.7. Leading Indicators (SPLD)

The scale of leading indicators was established by [76], and consist of four items which were used to assess the leading indicators at the workplace of safety performance. This scale’s purpose is to drive and measure activities carried out to prevent and control injury, such as safety audits, behavior, and attitude at the workplace. The items include the following: (1) “formal occupational health and safety audits at regular intervals are a normal part of our workplace”, (2) “everyone at this workplace appreciates ongoing occupational health and safety improvement in this workplace”, (3) “health and safety are important as product quality in the way the work is done”, and (4) “workers and supervisors have the information they need to work safely”.

#### 3.2.8. Lagging Indicators (SPLG)

In this study, the lagging indicator was measured by five items from the lagging indicator questionnaire by [77]. This scale aims to measure the effectiveness of a safety program after accidents have happened at the workplace, such as incidents and injuries. The items include the following: (1) “operating procedures are followed during start-up operations of the unit”, (2) “procedures are followed during emergency shutdown of the unit”, (3) “deviations are recorded from the written procedures during normal plant operations”, (4) “procedures are concise and clear”, and (5) “procedures dealing in different activities are incomplete”.

### 3.3. Sampling and Study Design

This study has developed a framework to demonstrate that good safety culture and safety are all related to safety performance, as shown in Figure 2. This framework has illustrated three important variables under investigation by the researcher (safety culture, psychosocial hazard, and safety performance). Psychosocial hazard was adapted as the mediator of the relationship between safety culture and safety performance. This study analyzed the mediating effect of psychosocial hazards between safety culture and safety performance in upstream oil and gas operations in Malaysia. This study also examined the different strategies for safety risk reduction and incident prevention by validating the framework constructs in the context of Malaysia. However, the proposed framework was assessed with the “Partial Least Square” approach using the software of Smart-PLS 3.2.7 [78]. The current study used a five-point Likert scale, which has also been used in other studies [79,80]. The survey questionnaire contained 30 items. The constructs variable’s structure is shown in Table 3.

To evaluate the structural models and measurement, we used the recommended two-staged analytical approaches [81]. G* Power version 3.1.9.2 was developed by [82], to calculate the suitable sample size. The required sample size of the study was 68, based on the 0.80 value recommended for “social and behavioral sciences”. The total sample size for the current research of 380 employees comfortably exceed the minimum sample size requirement. The current study had more participants than the optimum sample size for Smart-PLS-SEM analysis, which is 100 [83].

### 3.4. Structural Equation Modeling (SEM)

SEM is a multivariate method for determining the validity of competing hypotheses and gathered samples concerning a concept or theory [84,85]. The two main approaches for SEM are partial least squares structural equation modeling (PLS-SEM), and covariance-based structural equation modeling (CB-SEM) [86,87,88]. Mackinnon [89] mentioned that PLS-SEM is more flexible than CB-SEM when it comes to describing the link between items and constructs for researchers. PLS-SEM functions admirably in any sample size, but it must meet the sample size’s minimal criteria, which allows for the development of variables with complex effects on certain components of the model. PLS-SEM is concerned with constructs or latent variables that can be used with both reflective and non-reflective (formative) measurement models. As a result, researchers commonly incorporate the SEM technique. The following are some of the benefits of using SEM: to begin, SEM may be used to approximate complex hypothesis models based on multiple observations [90,91].

SEM is effective, particularly for very complicated models with large numbers of hidden variables and indicators. As a result, it seeks to obtain models that are as sparse as possible [91,92]. Many domains of social science studies, such as construction, industry, hotel management, competitive performance [93,94], the environment, and organization have successfully used SEM [95].

Finally, the PLS-SEM method was used to assess the four hypotheses that were proposed in this study. Bauer and Baumeister [96] indicated that variance inflation factor (VIF) was used to examine multicollinearity difficulties to evaluate multicollinearity. This was accomplished by using the Smart-PLS v3.2.1 tool to evaluate the measurement model’s fitting and path analysis [97]. Harman’s single factor was tested using SPSS version 25.0 software to test common technique bias.

## 4. Results

According to the guidelines provided in Equation (1), the GoF value was measured, to confirm the validity model of PLS-SEM. According to [98] who mentioned that the closer the GoF is to 1, the better the model in consideration fits will be GoF = (0 < GoF > 1). The model’s GoF was calculated precisely using the following formula:

Equation (1) describes the model’s GoF value:

(1) $$\mathrm{GoF}\;=\sqrt{R^{2}}\times \sqrt{\mathrm{AVE}}.$$

According to Table 4, the model of GoF showed that the average of R^2^ is 0.596, and that the mean AVE is 0.659. Therefore, based on the goodness of fit values, it is considered as a higher value of GoF.

### 4.1. Reliability Analysis

The measured model was evaluated using reliability and validity tests. During the reliability test, four indicators were examined: i.e., standardized indicator loadings (SIL), Cronbach’s Alpha (CA), Composite Reliability (CR), and Average Variance Extracted (AVE). All the values of CA [99], CR [100], and SIL [101] must be greater than ≥0.70. Every construct of AVE value should be higher than 0.5 [102,103]. For target constructions, the $R^{2}$ should be acceptable, with slandered values of (weak = 0.25, medium = 0.50, higher = 0.75). Additionally,$\;R^{2}$ explains the variance in the endogenous variable explained by the exogeneous variables. Table 5 displays the reliability and $R^{2}$ test results.

From the above table, the values of SIL were from 0.700 to 0.892 (≥0.700), and the CA values were from (0.863–0.906) above 0.700, the CR values were from 0.901 to 0.923 (>0.700), and the AVE values ranged from 0.574 to 0.647 (>0.700) [104]. Therefore, the $R^{2}$ value was from 0.208 up to 0.357 (≥0.10) according to Falk and Miller [105].

### 4.2. Discriminant Validity

According to Fornell and Larcker [106], the assessment of heterotrait-monotrait ratio of correlations was used to complete the discriminant validity test. In comparison to the Fornell-Larcker criterion and partial cross-loadings, the heterotrait-monotrait ratio (HTMT) test of correlations is preferable. As a result, the assessed model’s results revealed strong reliability, convergence validity, and discriminant validity and confirmed that the constructs were statistically diverse, as shown in Table 6.

### 4.3. Structural Model

#### 4.3.1. Direct Effect

Eight hypotheses were evaluated for hypotheses testing; three of them were tested as direct relationships, and the others were tested mediated relationships. One construct was used as an independent variable under (safety culture), one dependent variable was safety performance in the conceptual framework model, and the psychosocial hazard was the mediating variable. A repeated indicator approach was utilized to estimate the latent variables for these higher-order constructs (safety culture and safety performance). An independent variable’s strong effect on a dependent variable was measured using effect size [107]. The effect size ranged as (0.02 small, 0.15 moderate, 0.35 strong) [108]. Therefore, the effect size (*f*^2^) can be concluded by employing the equation recommended by Cohen (1988) [109], in accordance with SmartPLS analysis:

$$\mathrm{Effect}\;\mathrm{size}\;\left(f2\right)=\frac{R_{\mathrm{included}}^{2}-R_{\mathrm{excluded}}^{2}}{1-R_{\mathrm{included}}^{2}}.$$

**Construct****R^2^ Included****R^2^ Excluded****R^2^ Included-R^2^ Excluded****1-R^2^ Included****F2****Result**SC0.2090.1840.0250.7910.032SmallPSH0.2090.1470.0620.7910.078Small

**Figure 3:**
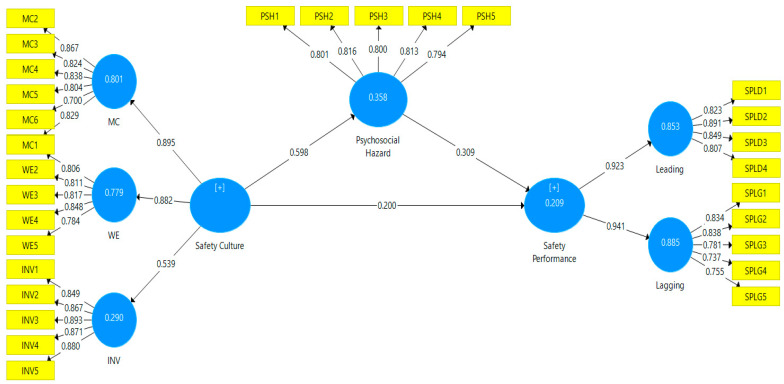
Measurement model outcomes.

Moreover, Table 7 shows that the effect sizes between factors were moderate to strong. Figure 3 depicts the results of measured model testing by using the Smart-PLS algorithm.

#### 4.3.2. Measurement Model Test

First, as with the original model estimate, we kept both missing values and the Smart-PLS-SEM algorithm settings. The following options for running the 5000 bootstrap samples were “Sign No Changes” and “Complete Bootstrapping.” Finally, the bias acceleration and correction method were utilized in the bootstrapping technique, and the advanced settings were performed as a one-tailed 0.05 significant level test. Table 7 shows that three hypotheses are supported (H1, H2, H3). All these hypotheses have a large impact size.

#### 4.3.3. Mediation Effect Measurement

The bootstrapping approach was utilized in this investigation to confirm the mediating impact [110]. For studies that try to analyze such indirect impacts, various scholars have utilized and advised this approach [111]. The outcomes of PLS provide the confidence intervals (CI) values of the indirect effect a*b, and when a 95% CI excludes zero, there is evidence of an indirect effect associating X and Y via the mediator with 95% confidence and then the mediation is established. Finally, the results of the indirect effect shown in Table 8 are more accurate and all mediating hypotheses were fully supported. Based on our findings, psychosocial hazards partially mediate between safety culture and safety performance.

## 5. Discussion

The main objective of this study was to assess the impact of safety culture on the workplace safety performance indicators, i.e., leading and lagging indicators with the mediation of psychosocial hazards. We investigated the impact of the psychosocial hazard on safety performance after developing direct hypotheses concerning safety culture and psychosocial hazard. It was proposed that a high level of safety culture would enable the provision of sufficient resources to contribute towards ensuring good safety performance by reducing employees psychosocial hazard, based on the social exchange theory [68,112,113]. As expected, psychosocial hazard mediated the relationship between safety culture and safety performance. It was obvious to find psychosocial hazard’s interplay between safety culture and safety performance. In doing so, by reducing psychosocial hazards for the workforce, organizations will allow their workers to invest their saved resources in learning and improving their safety habits. Our research contributes to the field of theory in a significant way. First, it confirmed that safety culture theory promotes individual safety performance by decreasing the psychosocial hazard [15,114]. In harmony with prior literature, safety culture dimensions such as management commitment, work environment, and involvement of workers collectively and positively impact safety performance [1]. Second, the findings emphasize the need to enhance the entire safety culture to promote the safety performance among employees [115]. Finally, safety performance was improved by reducing their indicators (leading and lagging), which can prevent any accident occurring during the workplace environment [71,116].

In the current study, four hypotheses were tested, three hypotheses were direct predictors, while the rest utilized psychosocial hazard as a mediating relationship between safety culture and safety performance. Furthermore, Table 7 indicates that the structure of the safety culture construct strongly predicts safety performance, with all of the hypotheses (H1, H2, H3, H4) being statistically significant.

Safety culture was calculated to form three viewpoints: management commitment, work environment, and involvement. Safety culture explored the employee’s understanding of the shared perception of safety preference and its impact on their performance as well as occupational health in the work environment. When employees are spending most of their time at their workplace, it can affect their psychosocial health through work strain and other occupational hazards. Organizations operating in the upstream oil and gas sector should improve workers’ productivity, wellbeing, and ensure their safety at the workplace nationwide in general. Therefore, this study found that, in (H1) the safety culture impacts the safety performance (β = 0.598, t = 14.619, *p* < 0.000). In the second construct, for (H2), psychosocial hazard has a significant effect on safety performance (β = 0.309, t = 4.22, *p* < 0.000), additionally, in hypothesis (H3), the study found that safety culture impacts on safety performance as well (β = 0.200, t = 2.881, *p* < 0.004). This result is consistent with findings from several previous research [44,67,117,118]. According to all of these research hypotheses, safety culture has a significant effect on safety performance and psychosocial hazards. When a third variable or construct is combined with other two similar constructs, a mediating influence is emerged [119].

The psychosocial hazard constructs mediated the relationship between safety culture and safety performance [120,121]. Table 8 results show a significant mediating effect of psychosocial hazard between the independent variable (IV) and dependent variable (DV). The results of the investigation thus validated the statistical significance of the hypothesis (H4). Preacher and Hayes [122] found that 0.277, 95% boot Cl (LL = 0.092, UL= 0.277) did not cross a 0 in the indirect effect (H4), indicating that there is a mediation. Therefore, the statistical findings indicate that there is a significant mediation impact between variables. Additionally, as shown in Table 7, there is a significant direct impact between SC (IV) and SP (DV) (β = 0.200, t = 2.881, *p* < 0.004). As a result, PSH mediates the relationship between SC and SP, so (H4) is strongly supported. Our findings of the study indicated that the psychosocial hazard mediated the relationship between safety culture and safety performance among the upstream oil and gas sector in Malaysia. This means that safety performance, such as leading and lagging is negatively impacted by safety culture [123,124]. Therefore, these contribute to safety performance through the psychosocial hazard, and this argument views the influence of safety culture on safety performance. As a result, these findings are consistent with those of earlier relevant investigations [125,126,127].

From a practical implementation perspective, organizations may promote the safety culture’s sub-dimensions to reduce psychosocial hazards at the workplace, thus possibly leading to the improvement in occupational health and safety at large. The workforce, which must face fewer psychosocial hazards, may perform safer by paying attention at large to the things at hand. It will also help managers to design interventions that can help achieve productivity and safety simultaneously.

## 6. Conclusions

In conclusion, safety culture has always been a common scene to determine the underlying and root causes of incidents. Research has been carried out by different researchers to identify the method in determining the safety, health, and environment culture in the organization, especially in the upstream petroleum industry as it is a high-risk industry where the consequences of incidents are generally high and impact the employees and environment. However, until today, there have been no satisfactory models developed that can measure the health, safety, and environment culture as a whole and, at the same time, identify the areas of improvement of the organization. This study examined the connection between the level of upstream operations of the oil and gas industry and employees who are the most vulnerable asset for any organization.

According to the findings and discussion above, this study examined the mediating of psychosocial hazards in the relationship between safety culture and safety performance. The results found that the relationship between safety culture and safety performance is significant. In addition, psychosocial hazards have been shown to impact safety performance significantly. Furthermore, the outcomes demonstrated the mediating effect of psychosocial hazard as a mechanism that investigates the relationship between safety culture and safety performance.

The purpose of focusing on upstream operation employees for the oil and gas industry is due to the reason that this sector has the highest rate of accidents/injuries in Malaysia. Upstream operations are also the most susceptible to safety incidents, and the employees work in the most harmful conditions [128], which makes them vulnerable to accidents and injuries at the workplace.

Our findings are based on the data that we have gathered from the Malaysian oil and gas upstream sector and mainly represent the local scenario; however, our findings can also be helpful if generalized for an international context. 

### Study Limitations

Despite the study’s accomplishments, the inherent limitations of the data collection approach are acknowledged. Further, our study is cross-sectional, whereas the longitudinal study is expected to produce more valuable insights. Moreover, our study represents a distinct section of global upstream operations in the oil and gas sector, although globally upstream operations operate on similar rules and procedures, thus findings of the research may not apply to all occupational settings or they work partially. The nature of participants is another limitation in our study as they are from a specific part of the population, thus adding research to a population that is more versatile/global can add more insights to the field of safety science. Our study is limited to the Malaysian culture context, and it needs to be validated in other contexts. Additional methods of data collection, such as self-evaluation, peer review, and diary observation, may be used in future studies to improve precision and boost the predictive power of study results. Safety climate and safety communication among oil and gas employees are two more fascinating topics that could be investigated in the future, to assess their potential to be translated to other occupational settings.

## Figures and Tables

**Table 1:** Safety culture definitions.

References	Safety Culture Definitions
[23]	Safety culture is considered the values, perceptions, attitudes, behaviors of individuals and groups that evaluate the commitment to health and safety management.
[24]	Culture interacts between people’s psychological and work behavior in the organization. Safety culture observes the efforts of organizational members and draws their attention towards daily safety improvement.
[15]	Safety culture is characterized as all elements of organizational culture that affect the behaviors and attitudes associated with increased or decreased risk.
[25]	To direct people’s activities toward risk, accidents, and prevention, it is required to share and understand all related definitions, experiences, and safety perceptions.
[26]	Employees’ decisions also depend on the organizational culture, which influences the overall company’s achievement.
[27]	Organizational culture in the workplace can be complex and comprehensive, yet ambiguous enough to be observed by the employees. In other words, if employees could not adapt to their organization’s culture, they will be thought of as outsiders.
[28]	A group’s safety culture evolves and changes through time as a result of changes in a variety of influencing elements such as leadership, management techniques, business environment, and formal and informal socialization processes.

**Table 2:** Demographic of the Respondents (N = 380).

Construct	Categories	Frequency	Responses %
Gender	Male	371	97.63
	Female	9	2.37
Age	20–29 Years	50	13.16
	30–39 Years	210	55.26
	40–49 Years	90	23.68
	50–59 Years	30	7.90
Work Experience	1–5 Years	102	26.84
	6–10 Years	127	33.42
	11–15 Years	45	11.84
	16–20	64	16.84
	21 Years and above	42	11.06
Marital Status	Single	62	16.32
	Married	295	77.63
	Divorced	23	6.05
Education	Graduate/Postgraduate	7	1.84
	College	53	13.94
	Secondary	312	82.11
	Primary	8	2.11

**Table 3:** Study instrument structured.

Constructs	Dimensions	No. of Items	References
Safety Culture		16	[73,74]
	Management Commitment (MC)	6	
	Work Environment (WE)	5	
	Involvement (INV)	5	
Safety Performance		9	[76,77]
	Leading (SPLD)	4	
	Lagging (SPLG)	5	
Psychosocial Hazard (PSH)		5	[75]

**Table 4:** Model of GoF.

Laten Variable	(AVE)	R Square
INV	0.761	0.29
Lagging	0.624	0.885
Leading	0.711	0.853
MC	0.66	0.801
Safety Culture	0.648	
Safety Performance	0.635	0.209
WE	0.575	0.779
Psychosocial Hazard	0.662	0.358
Average The goodness of Fit GoF	0.6585	0.5964 0.61

$R^{2}\;$endogenous constructs, AVE (Average variance extracted), GoF (Goodness of Fit) beseline value (small = 0.1, medium = 0.25, high = 0.36).

**Table 5:** PLS validity, reliability, and $R^{2}$ value.

Constructs	Path Relationship	SIL	CA	CR	AVE	Value	$R^{2}\;\mathrm{LEP}$
	SC		**0.907**	**0.920**	**0.635**	-	-
	MC1←SC	0.829					
	MC2←SC	0.867					
	MC3←SC	0.824					
	MC4←SC	0.838					
	MC5←SC	0.804					
	MC6←SC	0.700					
	WE1←SC	0.806					
	WE2←SC	0.811					
	WE3←SC	0.817					
	WE4←SC	0.848					
	WE5←SC	0.784					
	INV1←SC	0.849					
	INV2←SC	0.867					
	INV3←SC	0.893					
	INV4←SC	0.871					
	INV5←SC	0.880					
	PSH		**0.864**	**0.902**	**0.648**	**0.209**	**Medium**
	PSH1←PSH	0.801					
	PSH2←PSH	0.816					
	PSH3←PSH	0.800					
	PSH4←PSH	0.813					
	PSH5←PSH	0.794					
	SP		**0.907**	**0.924**	**0.575**	**0.358**	**Substantial**
	LD1←SP	0.823					
	LD2←SP	0.891					
	LD3←SP	0.849					
	LD4←SP	0.807					
	LG1←SP	0.834					
	LG2←SP	0.838					
	LG3←SP	0.781					
	LG4←SP	0.737					
	LG5←SP	0.755					

SIL: Standardized indicator loadings, CA: Cronbach’s Alpha, CR: Composite Reliability, AVE: average variance extracted, LEP: Level Explanatory power, SC: Safety Culture, MC: Management commitment, WE: Work Environment, INV: Involvement, PSH: Psychosocial Hazard, SP: Safety Performance, LD: Leading, LG: Lagging.

**Table 6:** Discriminant validity results.

	INV	LG	LD	MC	PSH	SC	SP	WE
INV	0.872							
LG	0.193	0.79						
LD	0.196	0.739	0.843					
MC	0.221	0.347	0.24	0.812				
PSH	0.61	0.429	0.365	0.432	0.805			
SC	0.539	0.406	0.303	0.589	0.598	0.654		
SP	0.208	0.419	0.329	0.319	0.429	0.385	0.759	
WE	0.241	0.396	0.278	0.754	0.429	0.285	0.366	0.814

**Table 7:** Direct Effect Results.

Hypothesis	H1	H2	H3
Path Relationship	SC→PSH	PSH→SP	SC→SP
Path coefficients β	0.598	0.309	0.2
Standard Error	0.041	0.073	0.069
T values	14.619	4.22	2.881
*p* values	0.000	0.000	0.004
Significance level	***	***	***
Results	Supported	Supported	Supported

Note: if = *p* > 0.05 (Not Significant), *** *p* < 0.05 (Significant), *** = *p* < 0.01 (Significant).

**Table 8:** Direct and indirect mediation results.

Mediation Effect	Path Coef	SD	*t*-Value	95% LL	95% UL	Results
SC-PSH-SP	0.185 **	0.047	3.946	0.092	0.277	Partial Mediation

Note: ** = *p* < 0.01, SD: Standard Deviation, LL: lower level, UL: upper level.

## Data Availability

Currently, we still working in this project, and we still need to use the data for further works and analysis. However, any researcher needs the data for further investigations can contact the corresponding author via email.
